# Sex and Gender-Related Differences in COVID-19 Diagnoses and SARS-CoV-2 Testing Practices During the First Wave of the Pandemic: The Dutch Lifelines COVID-19 Cohort Study

**DOI:** 10.1089/jwh.2021.0226

**Published:** 2021-12-16

**Authors:** Aranka Viviënne Ballering, Sabine Oertelt-Prigione, Tim C. olde Hartman, Judith G.M. Rosmalen

**Affiliations:** ^1^ Interdisciplinary Center Psychopathology and Emotion Regulation (ICPE), University Medical Center of Groningen, University of Groningen, Groningen, the Netherlands.; ^2^ Department of Primary and Community Care, Radboud University Medical Center, Nijmegen, the Netherlands.; ^1^ Department of Epidemiology, University Medical Center Groningen, University of Groningen, Groningen, The Netherlands.; ^2^ Faculty of Economics and Business, University of Groningen, Groningen, The Netherlands.; ^3^ Aletta Jacobs School of Public Health, Groningen, The Netherlands.; ^4^ Department of Genetics, University Medical Center Groningen, University of Groningen, Groningen, The Netherlands.; ^5^ Department of Genetics, Center of Development and Innovation, University Medical Center Groningen, University of Groningen, Groningen, The Netherlands.; ^6^ Department of Psychiatry, University Medical Center Groningen, University of Groningen, Groningen, The Netherlands.

**Keywords:** COVID-19, SARS-CoV-2, sex differences, gender equity

## Abstract

***Background:*** Although sex differences are described in Coronavirus Disease 2019 (COVID-19) diagnoses and testing, many studies neglect possible gender-related influences. Additionally, research is often performed in clinical populations, while most COVID-19 patients are not hospitalized. Therefore, we investigated associations between sex and gender-related variables, and COVID-19 diagnoses and testing practices in a large general population cohort during the first wave of the pandemic when testing capacity was limited.

***Methods:*** We used data from the Lifelines COVID-19 Cohort (*N* = 74,722; 60.8% female). We applied bivariate and multiple logistic regression analyses. The outcomes were a COVID-19 diagnosis (confirmed by SARS-CoV-2 PCR testing or physician's clinical diagnosis) and PCR testing. Independent variables included among others participants' sex, age, somatic comorbidities, occupation, and smoking status. Sex-by-comorbidity and sex-by-occupation interaction terms were included to investigate sex differences in associations between the presence of comorbidities or an occupation with COVID-19 diagnoses or testing practices.

***Results:*** In bivariate analyses female sex was significantly associated with COVID-19 diagnoses and testing, but significance did not persist in multiple logistic regression analyses. However, a gender-related variable, being a health care worker, was significantly associated with COVID-19 diagnoses (OR = 1.68; 95%CI = 1.30–2.17) and testing (OR = 12.5; 95%CI = 8.55–18.3). Female health care workers were less often diagnosed and tested than male health care workers (OR_interaction_ = 0.54; 95%CI = 0.32–0.92, OR_interaction_ = 0.53; 95%CI = 0.29–0.97, respectively).

***Conclusions:*** We found no sex differences in COVID-19 diagnoses and testing in the general population. Among health care workers, a male preponderance in COVID-19 diagnoses and testing was observed. This could be explained by more pronounced COVID-19 symptoms in males or by gender inequities.

Globally, males and females are infected with Severe Acute Respiratory Syndrome-Coronavirus-2 (SARS-CoV-2), the virus responsible for Coronavirus Disease 2019 (COVID-19), at equal rates.^1,2^ However, sex differences are found in multiple other aspects of COVID-19. Males experience higher rates of hospitalizations, intensive care unit admission, and COVID-19-related deaths.^1,3^ These differences can be partly explained by biological sex differences, for example in innate and humoral immune responses^4^ or in rates of pre-existing somatic comorbidities, such as cardiovascular disease, which associate with a poor COVID-19 prognosis.^5–7^ In addition to these sex-specific differences, gender-related factors associate with the course of SARS-CoV-2 infection. Gender is the embodiment of different roles, behaviors, identities, and relationships by individuals according to societal norms, which result in different expectations, opportunities, and experiences.^8^ It can modify risk factor distribution and exposure patterns.^3^ For example, women constitute the majority of the health workforce worldwide, and are more likely to work in the service industry and contact professions.^9^ A recent study, including 99,795 health care workers and 2,035,395 community individuals showed that health care workers had a 3.4-fold greater risk of infection with SARS-CoV-2, compared with the general population.^10^ Epidemiological data about COVID-19 are thus influenced by sex differences, and gendered health-related behaviors and roles. Disentangling the contribution of these factors is complex as the presence of multilayered interactions have to be assumed.^11^

Gender can also affect access to and uptake of diagnostic measures. A Canadian study, including 233,566 individuals that received SARS-CoV-2 tests, demonstrated that 64.3% of the conducted tests were performed in women.^12^ Two other Canadian general population studies, including 409,207 and 4,240 individuals showed no difference or a small skewing toward the uptake of tests in women, respectively.^13^

Most medical research on sex differences in COVID-19 is conducted in clinical populations, which represent a fraction of the affected population. Hence, we are currently unable to formulate health care system-wide implications and recommendations based on these data.^14^ Additionally, most clinical research and large-scale epidemiological research neglects the possible influence of gender. Few previous studies accounted for participants' occupation and the gendered aspect hereof. Given the significant overrepresentation of women in the health care workforce and other professions that require being in the proximity of other individuals, such as those in education, it is important to adjust for these factors when assessing sex and gender-related differences in health outcomes.

Both sex- and gender-related risk factors are known to unequally affect men's and women's health and access to health care.^15,16^ First, obtaining insights into possible influences of sex and gender during the first wave of the COVID-19 pandemic is pivotal for understanding health-related inequities between women and men during times of health-resource scarcity. Second, these insights may bear important policy implications for future disaster preparedness. Third, they might be especially relevant for low- and middle-income countries where health-resource scarcity during an epidemic or pandemic may persist longer due to pre-existing resource constraints. Lastly, to inform public health policies, conclusions from clinical research studies do not suffice and general population studies should be performed as well.

Therefore, we investigated the associations between sex and gender-related factors with COVID-19 diagnoses and testing practices in the Lifelines COVID-19 Cohort study, which includes 74,722 unique participants from the general population of the North of the Netherlands. We hypothesize that both female sex and feminine gender factors associate with a COVID-19 diagnosis and SARS-CoV-2 testing practices.

## Methods

### Data source

This study is based on data collected within the Dutch Lifelines Cohort Study and its digital add-on study, the Lifelines COVID-19 cohort. The former is a multidisciplinary prospective population-based cohort study examining in a unique three-generation design the health and health-related behaviors of 167,729 persons living in the North of The Netherlands. It employs a broad range of investigative procedures in assessing the biomedical, sociodemographic, behavioral, physical, and psychological factors, which contribute to the health and disease of the general population, with a special focus on multimorbidity and complex genetics.

We included data of 13 consecutive Lifelines COVID-19 measurements from March 2020 to August 2020. Initially questionnaires for continuous data collection were send out weekly; from June 2020 onward data were collected at biweekly intervals. Participants of the Lifelines COVID-19 cohort are recruited from the Lifelines population cohort and the Lifelines NEXT birth cohort. The Lifelines Cohort Study is performed according to the principles of the Declaration of Helsinki. The Lifelines Cohort Study is approved by the Medical Ethics Committee of the University Medical Center Groningen (number: 2007/152). All participants provided written consent. Extensive information on the cohorts, design considerations, and recruitment procedures is provided elsewhere.^17–20^

### COVID-19 and the testing regime in the North of the Netherlands

The first confirmed COVID-19 case in the Netherlands was reported on February 27, 2020. A month later, the number of new cases diagnosed per day reported by the municipal health services was 1,178. The number of deaths related to COVID-19 had risen as well, with a peak of 178 deaths per day on April 2, 2020.^21^ The steep rise in cases and case fatalities prompted the Dutch government to announce a nation-wide lockdown on March 15, 2020.

In the Northern provinces of the Netherlands the COVID-19 outbreak followed a different pattern than in other regions of the country. The first COVID-19 cases were reported on March 1, 2020, March 10, 2020, and March 11, 2020 in the Northern provinces of Drenthe, Friesland, and Groningen, respectively.^20^ Up until June 9, 2020, merely 3.1%, 2.7%, and 2.0% of the national cumulative SARS-CoV-2 infections, hospitalizations, and COVID-19-related deaths were reported in the North of the Netherlands, respectively. Additional information on the COVID-19 outbreak, its facilitators, and barriers in the Northern Dutch provinces can be found elsewhere.^20^

Notably, the testing regime in the Netherlands was restricted during the first wave of the COVID-19 pandemic, as a shortage of reagents occurred during the first months of the pandemic.^22^ This meant that health care workers and severely ill patients with a suspected SARS-CoV-2 infection were prioritized in testing procedures. The limited availability of SARS-CoV-2 testing equipment for the general population persisted until August 2020.

### Participants

Participants over 18 years of age completed digital questionnaires on multiple topics, including but not limited to demographics, occupation, physical and mental health, and adherence to COVID-19 guidelines. The questionnaires register the participants' municipally registered sex, which generally corresponds to biological sex at birth, hence we refer to the participants as male and female. The questionnaire does not include a specific question about gender identity. Of the 74,722 unique participants (18–94 years of age) included in this study, 61,584 (82.4%) unique participants were included during the first three measurements (Supplementary Appendix A1). In total, 30,326 (40.6%) participants completed 10 or more questionnaires. Additional information about the study population is shown in Table 1.We did not find any indication for relevant systemic attrition: no meaningful associations between potential predictors of infection or testing and attrition rates existed. Similarly, proportions of missing data did not differ meaningfully between groups (*e.g.,* male/female or infected/noninfected).

**Table 1.** Characteristics of the Study Population

	Male participants (*N* = 29,273; 39.2%)	Female participants (*N* = 45,449; 60.8%)
Age in years, mean (SD)	55.4 (12.9)	52.7 (12.9)
Educational attainment, *N* (%)		
Low	4,209 (14.4)	5,113 (11.2)
Medium	13,351 (45.6)	23,820 (52.4)
High	11,319 (38.7)	15,768 (34.7)
PCR test conducted, *N* (%)	191 (0.7)	602 (1.3)
Positive PCR test, *N* (%)	52 (0.2)	131 (0.3)
Positive doctor's diagnosis, *N* (%)	322 (1.1)	661 (1.5)
COVID-19 diagnosis^a^, *N* (%)	355 (1.2)	751 (1.7)
Hospitalized for a COVID-19 diagnosis, *N* (%)	13 (0.0)	<10 (0.0)
Chronic disease, *N* (%)		
Yes	5,896 (20.1)	10,526 (23.2)
No	16,409 (56.1)	24,469 (53.8)
Smoking, *N* (%)		
Yes	2,896 (9.9)	3,979 (8.8)
No	25,825 (88.2)	40,634 (89.4)
Alcohol, *N* (%)		
Yes	19,602 (67.0)	23,010 (50.6)
No	9,089 (31.0)	21,562 (47.4)
Precautions taken, *N* (%)		
Frequently washing hands or use of disinfectant	27,293 (93.2)	43,542 (95.8)
Social distancing	28,095 (96.0)	43,831 (96.4)
Avoid use of public transport	20,092 (68.6)	33,474 (73.7)
Covering nose and mouth in public	2,770 (9.5)	4,126 (9.1)
Household includes a child, *N* (%)	7,669 (26.2)	12,145 (26.7)
Household includes another adult, *N* (%)	15,547 (53.1)	22,780 (50.1)
Household includes an elderly person, *N* (%)	7,715 (26.4)	13,045 (28.7)
Profession that requires contact, *N* (%)		
No	15,416 (52.7)	15,782 (34.7)
Yes, but no education or health professional	2,132 (7.3)	7,062 (15.5)
Yes, as an education professional	1,418 (4.8)	3,454 (7.6)
Yes, as a health care professional	670 (2.3)	3,034 (6.7)
Not reported	9,562 (32.9)	16,146 (35.5)

^a^ Defined as receiving either a positive SARS-CoV-2 PCR test, or a positive clinician's diagnosis.

### Variables and statistical analyses

Descriptive statistics are provided as absolute numbers with concomitant percentages. If appropriate, means with standard deviations are provided. Data were examined for normality using q-q plots and histograms.

The outcome variables included participants' receipt of a COVID-19 diagnosis and test for SARS-CoV-2. We defined a COVID-19 diagnosis as either a self-reported positive SARS-CoV-2 PCR test or a self-reported clinical diagnosis of COVID-19 by a physician based on participants' symptoms. We provide an overview of the population's characteristics stratified by diagnosis method (Supplementary Appendix A2).

All independent variables were self-reported and the entry given during the first completed questionnaire was included in analyses. The presence of chronic diseases covered a range of somatic diseases, including but not limited to cardiovascular, autoimmune, and neurological diseases (Supplementary Appendix A3). Age and biological sex were derived from the municipal registration database. Participants' educational level was derived from the Lifelines Cohort Study data and defined as described earlier.^23^ Participants' type of contact profession was derived from both the Lifelines Cohort Study data and the Lifelines COVID-19 Cohort. The former provided data, up until 2018, on whether participants were health care or educational professionals (ISCO submajor group 22 and 23, respectively), while the COVID-19 Cohort provided information about whether participants had a contact profession.

To identify whether sex- and gender-related variables associate with COVID-19 diagnoses, we conducted multiple logistic regression analyses. Participants' sex, age in years, educational level, presence of chronic somatic diseases, smoking status, adherence to mitigation guidelines, household composition, working place, and (contact requiring) occupation were included as independent variables. We included interaction terms between sex and the presence of a chronic disease, and between sex and occupation to assess whether the association between the respective independent variables and outcome differed per sex. Participants with missing data on independent variables were excluded listwise.

We conducted a similar multiple logistic regression analysis with SARS-CoV-2 testing as an outcome, but with fewer independent variables due to fewer testing events. Participants' biological sex, age in years, educational attainment, presence of chronic somatic disease, smoking status, household composition, working place, and (contact requiring) occupation were included as independent variables. Interaction terms between sex and the presence of chronic disease, and sex and occupation were included in the analyses as well.

To assess whether participants' age in years fulfilled the linearity assumption of multiple logistic regression analyses, we divided the variable into quartiles and assessed whether the odds of being diagnosed or tested were monotonically changing. We maintained a two-sided α-value, corrected for multiple comparisons, of 0.002 (0.05/23, 19 predictors and 4 sex-by-variable interaction terms within a family of tests). IBM SPSS v. 25 was used to perform analyses.

## Results

In total, 544,077 questionnaires were completed by 74,722 unique participants (60.8% female). Table 1 provides an overview of the characteristics of the study population.

### COVID-19 diagnosis

In bivariate analyses female sex associated with a COVID-19 diagnosis: females had 1.37 (95%CI = 1.21–1.55) times the odds of males to be diagnosed with COVID-19 (Supplementary Appendix A4). However, Table 2 shows that this association did not persist if adjusted for working a contact profession and additional variables (OR = 0.94; 95%CI = 0.81–1.09). Working in a health care profession was found to be positively associated with a COVID-19 diagnosis, (OR = 1.68; 95%CI = 1.30–2.17). In female participants the association between being a health care worker and a COVID-19 diagnosis was statistically different (OR = 1.84; 95%CI = 1.61–2.12) from that in male participants (OR = 2.69; 95%CI = 1.66–4.38). The interaction term shows female health care workers were diagnosed less often than their male counterparts (OR_interaction_ = 0.54; 0.32–0.92).

**Table 2.** Associations Between Predictors and COVID-19 Diagnosis

	Total population (*N* = 74,722)	Male participants (*N* = 29,273)	Female participants (*N* = 45,449)
	OR (95% CI)	OR (95% CI)	OR (95% CI)
Female sex	0.94 (0.81–1.09)	n.a.	n.a.
Age	0.99 (0.99–1.00)	1.01 (0.99–1.02)	0.99 (0.98–1.00)
Educational attainment			
Low	1.00 (ref)	1.00 (ref)	1.00 (ref)
Medium	0.94 (0.72–1.23)	0.78 (0.53–1.16)	1.06 (0.74–1.53)
High	0.68 (0.52–0.91)	0.69 (0.45–1.05)	0.69 (0.47–1.03)
Chronic disease present	**1.34 (1.15–1.55)^a^**	1.18 (0.91–1.52)	**1.48 (1.36–1.62)**
Smoking	0.92 (0.71–1.19)	0.86 (0.57–1.32)	0.89 (0.76–1.04)
Frequent handwashing and use of disinfectant	0.72 (0.45–1.15)	0.76 (0.42–1.38)	0.69 (0.32–1.47)
Social distancing	**0.22 (0.11–0.44)**	**0.26 (0.09–0.79)**	**0.21 (0.13–0.35)**
Avoidance of public transport	1.16 (0.98–1.37)	1.14 (0.87–1.49)	1.01 (0.92–1.11)
Covering nose and mouth in public	**1.61 (1.33–1.97)**	**1.53 (1.10–2.13)**	**1.99 (1.78–2.22)**
Household members ≤18 years	0.98 (0.83–1.16)	0.96 (0.73–1.27)	1.00 (0.91–1.09)
Household members 19–59 years	**0.51 (0.42–0.60)**	**0.43 (0.32–0.59)**	**0.79 (0.71–0.89)**
Household members ≥60 years	**0.76 (0.61–0.94)**	**0.63 (0.42–0.93)**	0.91 (0.79–1.03)
Working from home	**1.51 (1.30–1.78)**	**1.68 (1.28–2.19)**	1.02 (0.93–1.13)
Contact profession			
No	1.00 (ref)	1.00 (ref)	1.00 (ref)
Yes, but not in education or health care	**1.22 (1.02–1.47)^b^**	**1.43 (1.04–1.98)**	**1.34 (1.12–1.49)**
Yes, in education	**1.38 (1.08–1.77)^c^**	1.07 (0.65–1.75)	**1.32 (1.13–1.53)**
Yes, in health care	**1.68 (1.30–2.17)^d^**	**2.69 (1.66–4.38)**	**1.84 (1.61–2.12)**

Bold values indicate significance of *p* < 0.002.

^a^ Sex-by-chronic disease interaction: OR = 1.17, 95%CI = 0.86–1.60.

^b^ Sex-by-contact profession interaction: OR = 0.91, 95%CI = 0.64–1.31.

^c^ Sex-by-education profession interaction: OR = 1.29, 95%CI = 0.75–2.20.

^d^ Sex-by-health care profession interaction: OR = 0.54, 95%CI = 0.32–0.92.

### SARS-CoV-2 testing

The bivariate analyses in Supplementary Appendix A4 show that female sex was statistically significantly associated with SARS-CoV-2 PCR testing (OR = 2.04; 95%CI = 1.74–2.41). However, as shown by Table 3, female sex was not significantly associated with SARS-CoV-2 testing upon adjustment for additional variables (OR = 1.04; 95%CI = 0.77–1.42). Health care workers had higher odds of being tested for SARS-CoV-2 (OR = 12.5; 95%CI = 8.55–18.3) than the reference category. Notably, female health care workers were less often tested than their male counterparts (OR_interaction_ = 0.53; 95%CI = 0.29–0.97).

**Table 3.** Associations Between Predictors and Receiving SARS-CoV-2 PCR Tests

	Total population (*N* = 74,722)	Male participants (*N* = 29,273)	Female participants (*N* = 45,449)
	OR (95%CI)	OR (95% CI)	OR (95% CI)
Female sex	1.04 (0.77–1.42)	n.a.	n.a.
Age	**0.97 (0.95–0.98)**	0.97 (0.95–1.00)	**0.96 (0.95–0.98)**
Educational attainment			
Low	1.00 (ref)	1.00 (ref)	1.00 (ref)
Medium	1.10 (0.63–1.93)	0.50 (0.19–1.30)	1.39 (0.67–2.89)
High	1.18 (0.66–2.10)	0.79 (0.31–2.04)	1.31 (0.62–2.79)
Chronic disease present	0.94 (0.70–1.26)^a^	0.85 (0.45–1.59)	0.96 (0.69–1.33)
Smoking	0.67 (0.39–1.16)	0.19 (0.03–1.35)	0.84 (0.47–1.49)
Household members ≤18 years	1.03 (0.78–1.36)	0.98 (0.54–1.80)	1.07 (0.78–1.47)
Household members 19–59 years	**0.57 (0.41–0.80)**	**0.46 (0.22–0.95)**	**0.60 (0.41–0.87)**
Household members ≥60 years	0.80 (0.53–1.21)	1.09 (0.45–2.62)	0.72 (0.45–1.16)
Working from home	**0.46 (0.33–0.66)^b^**	1.13 (0.62–2.06)	**0.30 (0.18–0.47)**
Contact profession			
No	1.00 (ref)	1.00 (ref)	1.00 (ref)
Yes, but not in education or health care	**2.86 (1.96–4.17)^c^**	**2.70 (1.26–5.80)**	**2.52 (1.59–3.99)**
Yes, in education	1.41 (0.70–2.83)^d^	1.89 (0.63–5.65)	1.16 (0.47–2.86)
Yes, in health care	**12.5 (8.55–18.3)^e^**	**21.4 (11.0–41.6)**	**10.1 (6.34–16.1)**

Bold values indicate significance of *p* < 0.002.

^a^ Sex-by-chronic disease interaction: OR = 1.04, 95%CI = 0.52–2.09.

^b^ Sex-by-working from home interaction: OR = 0.27, 95%CI = 0.13–0.53.

^c^ Sex-by-contact profession interaction: OR = 2.28, 95%CI = 1.10–4.72.

^d^ Sex-by-educational profession interaction: OR = 0.38, 95%CI = 0.10–1.42.

^e^ Sex-by-health care profession interaction: OR = 0.53, 95%CI = 0.29–0.97.

n.a., not applicable.

## Discussion

This study is the first large epidemiological study in the general population that assesses sex and gender-related differences in COVID-19 diagnosis and SARS-CoV-2 testing in the first wave of the COVID-19 pandemic in the Netherlands. In bivariate analyses we found that females had higher odds than males on receiving a SARS-CoV-2 PCR and a COVID-19 diagnosis. However, upon adjustment for additional covariates, these sex differences did not persist, which is in contrast with our hypothesis. Health care workers received significantly more tests and COVID-19 diagnoses than other employee groups, with male health care workers receiving significantly more tests and COVID-19 diagnoses than female health care workers.

### Strengths and limitations

The principal strength of this study was that the Dutch Lifelines Cohort is a large and already established cohort study. Therefore, information about participants was registered before the COVID-19 pandemic. This allowed us to include more information in our analyses than the Lifelines COVID-19 questionnaires. Another strength is that data were collected during the first wave of the COVID-19 pandemic, during which a scarcity in SARS-CoV-2 PCR tests occurred, allowing for more pronounced appearance of potential biases.

However, our study was limited by the relatively few COVID-19 cases in the North of the Netherlands. Moreover, we considered participants with a self-reported physician's COVID-19 diagnosis based on symptoms as COVID-19 positive, while a recent study shows that symptoms only moderately associate with a positive SARS-CoV-2 PCR test.^24^ Therefore, as physicians diagnosed patients with COVID-19 based on their symptoms, we may have overestimated the proportion of COVID-19-positive participants (*i.e.,* participants with detectable SARS-CoV-2) in our study population. We also included one's profession as a gender-related variable, but we did not account for other gender-related variables, for example unpaid (child) care responsibilities.

Furthermore, health care workers also differ in their professional tasks and disciplinary expertise, from intensivists to home nurses for example, thus displaying a range of occupational risk for COVID-19. Similarly, health care workers may have different hierarchical positions in the hospital, which may associate to the probability of a participant's uptake of a SARS-CoV-2 test. Additionally, participants may have changed occupation or retired between completing the occupational questionnaire up to 2018 in the Dutch Lifelines Cohort and the Lifelines COVID-19 cohort questionnaires. Lastly, as the presence and severity of COVID-19-related symptoms may be worse in men, possibly due to a less adequate innate and humoral immune response,^25^ this may have introduced a male bias in the access to SARS-CoV-2 testing as this often depends on the presence of symptoms.

### Comparison to literature

Although bivariate analyses showed that females in the general population were more often diagnosed with COVID-19 than males, these sex differences did not persist in multiple regression analyses. This is in line with previous research, including a recent meta-analysis of 3,111,714 cases, demonstrating that females and males have confirmed COVID-19 diagnoses at equal rates.^1,25,26^

The association between being a health care worker and receiving a COVID-19 diagnosis, in contrast, differed significantly between females and males: male health care workers had higher odds of a COVID-19 diagnosis than female counterparts. This seems to contradict earlier Dutch and Canadian research that focused on confirmed and suspected SARS-CoV-2 infection among health care workers. In these studies, 83.3% and 81.7% of the COVID-19 cases in health care workers were female, respectively.^27,28^ However, these studies did not adjust for the overrepresentation of females in the population of health care workers.

In contrast, a large study including 10,034 health care workers from the United Kingdom showed that male staff had an increased risk (OR = 1.19; 95%CI = 1.01–1.40) of SARS-CoV-2 IgG seropositivity, and likely had been infected with SARS-CoV-2, compared with female staff.^29^ Our finding also explains the apparent discrepancy between the observed equal infection rates in men and women,^1^ and the increased risk of health care workers, of which the majority are women^10^: although the odds of a COVID-19 diagnosis are increased in health care workers, especially male health care workers appear to be at risk.

In initial bivariate analyses we found that females had higher odds than males for SARS-CoV-2 testing. This is in line with descriptive Canadian studies that assessed sex-disaggregated data on PCR testing.^12,13^ However, upon adjustment for additional variables, such as occupation, the identified sex difference was no longer statistically significant.

In health care workers, this pattern was fully revised with male health care workers being at significantly higher odds of SARS-CoV-2 testing than female health care workers. Several studies argue that sex- and gender-related factors associate with male-biased patterns of access to diagnostic measures,^30,31^ which is in line with our study. We identified more testing in male than female health care workers, which might be related to symptoms being possibly more pronounced in males than in females.^32,33^ Moreover, gendered work segregation might also play an important role. In fact, a larger proportion of male health care workers are employed by hospitals, and not in domestic care or elderly care, where access to personal protective equipment and testing equipment was limited potentially resulting in not only undertesting, but also underdiagnosis.^34^

### Implications for practice and policy

Conclusively, bivariate analyses demonstrate sex differences in COVID-19 diagnoses and SARS-CoV-2 testing during the first wave of the pandemic in the general population, but these do not remain upon adjustment for additional covariates. Although a male preponderance in COVID-19 diagnoses and SARS-CoV-2 testing was found among health care workers, this may be related to the more pronounced COVID-19 symptoms and worse prognosis in males, respectively. Gender-related factors, such as occupation were found to be relevant in COVID-19 diagnoses and SARS-CoV-2 testing.

Therefore, further research could focus on the independent effects of sex and gender-related factors, besides occupation, on uptake of SARS-CoV-2 tests and COVID-19 diagnoses. For example, it should be investigated in detail whether the increased male test uptake among health care workers is due to more males working in hospital settings, in which more testing equipment was available than in female-dominated extramural and domestic care settings.

Moreover, the situation experienced during the first wave of the pandemic in the Netherlands may mimic a more persistent situation in low- and middle-income countries. Therefore, research in low- and middle-income countries could assess whether a comparable pattern (*i.e.,* predominant testing in male health care workers) exists, which may inform policy-making decisions on health-related gender equality.

Additionally, research should focus on whether a sex/gender bias remains once an open-to-all testing policy is in place, as this would point toward differences in willingness to test, instead of toward limitations in availability of tests. For policymakers, this study implies that conclusions drawn on sex and gender biases in COVID-19 diagnoses and testing from clinical studies should be interpreted with caution, as these cannot be readily extrapolated to other (care) settings, such as the general population or primary and elderly care. Overall, the identified bias in testing procedures should be evaluated on a larger scale to assess its justification or remove its inherent bias.

## Supplementary Material

Supplemental data

Supplemental data

Supplemental data

Supplemental data

## Supplementary Material

Supplementary Appendix A1

Supplementary Appendix A2

Supplementary Appendix A3

Supplementary Appendix A4
