# Supplementary material for: Sex and Gender-Related Differences in COVID-19 Diagnoses and SARS-CoV-2 Testing Practices During the First Wave of the Pandemic: The Dutch Lifelines COVID-19 Cohort Study
Source: J Womens Health (Larchmt). 2021 Dec 16;30(12):1686–92. doi: 10.1089/jwh.2021.0226 (PMC8721498; doi:10.1089/jwh.2021.0226)
Supplement: Supplemental data [file Supp_Appendix1.docx]

**Appendix A: Participants per measurement, stratified by sex**

|  | **Participants, N (%)** | | **New unique participants, N (%)** | |
| --- | --- | --- | --- | --- |
|  | **Male** | **Female** | **Male** | **Female** |
| **Measurement 1** | 20,571 (38.7) | 32,556 (61.3) | 20,571 (38.7) | 32,556 (61.3) |
| **Measurement 2** | 19,591 (38.7) | 31,030 (61.3) | 2,049 (40.6) | 2,992 (59.4) |
| **Measurement 3** | 19,152 (38.7) | 30,322 (61.3) | 960 (41.5) | 1,352 (58.5) |
| **Measurement 4** | 18,295 (38.8) | 28,892 (61.2) | 467 (42.3) | 637 (57.7) |
| **Measurement 5** | 17,651 (38.9) | 27,712 (61.1) | 353 (42.0) | 488 (58.0) |
| **Measurement 6** | 16,629 (38.8) | 26,178 (61.2) | 182 (40.1) | 272 (59.9) |
| **Measurement 7** | 16,647 (38.8) | 26,203 (61.2) | 3,590 (39.8) | 5,435 (60.2) |
| **Measurement 8** | 15,060 (39.4) | 23,126 (60.6) | 194 (42.4) | 264 (57.6) |
| **Measurement 9** | 13,726 (39.0) | 21,444 (61.0) | 152 (39.5) | 233 (60.5) |
| **Measurement 10** | 12,536 (38.1) | 20,408 (61.9) | 97 (31.9) | 207 (68.1) |
| **Measurement 11** | 13,442 (38.3) | 21,617 (61.7) | 205 (36.6) | 355 (63.4) |
| **Measurement 12** | 13,892 (38.4) | 22,242 (61.6) | 253 (41.3) | 359 (58.7) |
| **Measurement 13** | 13,558 (38.6) | 21,597 (61.4) | 200 (40.1) | 299 (59.9) |
| **Total** | 210,750 (38.7) | 333,327 (61.3) | 29,273 (39.2) | 45,449 (60.8) |
