# Supplementary material for: Sex and Gender-Related Differences in COVID-19 Diagnoses and SARS-CoV-2 Testing Practices During the First Wave of the Pandemic: The Dutch Lifelines COVID-19 Cohort Study
Source: J Womens Health (Larchmt). 2021 Dec 16;30(12):1686–92. doi: 10.1089/jwh.2021.0226 (PMC8721498; doi:10.1089/jwh.2021.0226)
Supplement: Supplemental data [file Supp_Appendix2.docx]

**Appendix B: Characteristics of participant’s infected with COVID-19, stratified by method of diagnosis.**

| **Characteristics of the population with COVID-19, split by method of diagnosis** | | | |
| --- | --- | --- | --- |
|  | | **Positive PCR test (N=183)** | **Physician’s COVID-19 diagnosis (N=983)** |
| **Sex, N (%)** | **Female** | 131 (71.6%) | 661 (67.2%) |
|  | **Male** | 52 (28.4%) | 322 (32.8%) |
| **Age, mean (SD)** | | 51.5 (10.3) | 51.2 (12.1) |
| **Education, N (%)** | **Low** | 17 (9.3%) | 87 (8.9%) |
|  | **Medium** | 100 (54.6%) | 500 (50.9%) |
|  | **High** | 34.3 (35.0%) | 386 (39.3%) |
| **Frequent handwashing and use of desinfectant, N (%)** | | 176 (96.2%) | 959 (97.6%) |
| **Social distancing, N (%)** | | 173 (94.5%) | 973 (99.0%) |
| **Avoidance of public transport, N (%)** | | 127 (69.4%) | 778 (79.1%) |
| **Covering nose and mouth in public, N (%)** | | 31 (16.9%) | 137 (13.9%) |
| **Contact profession, N (%)** | **No** | 61 (33.3%) | 476 (48.4%) |
|  | **Yes** | 61 (33.3%) | 217 (22.1%) |
|  | **Yes, in education** | 11 (6.0%) | 96 (9.8%) |
|  | **Yes, in healthcare** | 28 (15.3%) | 74 (7.5%) |
| **Smoking, N(%)** | | <10 (<5.0%) | 86 (8.7%) |
