# Supplementary material for: Sex and Gender-Related Differences in COVID-19 Diagnoses and SARS-CoV-2 Testing Practices During the First Wave of the Pandemic: The Dutch Lifelines COVID-19 Cohort Study
Source: J Womens Health (Larchmt). 2021 Dec 16;30(12):1686–92. doi: 10.1089/jwh.2021.0226 (PMC8721498; doi:10.1089/jwh.2021.0226)
Supplement: Supplemental data [file Supp_Appendix3.docx]

**Appendix C: Adjustment for somatic diseases**

| **Overarching disease type** | **Examples given in Lifelines questionnaire^a^** |
| --- | --- |
| **Cardiovascular disease** | High blood pressure |
|  | Heart attack |
|  | Narrowing of the arteries in the legs |
|  | Stroke/TIA |
|  | Other heart and/or coronary diseases |
| **Lung disease** | Asthma |
|  | COPD |
|  | Chronic bronchitis |
| **Liver disease** | Cirrhosis |
| **Kidney disease** | Reduced kidney function |
| **Diabetes** | Diabetes mellitus Type 1 |
|  | Diabetes mellitus Type 2 |
| **Chronic muscle disease** | MS |
| **Auto-immune illness** | Celiac disease |
|  | Inflammatory bowel disorder |
|  | Rheumatoid Arthritis |
|  | Lupus |
| **Cancer** | Any form of cancer |
| **Neurological disease** | Dementia |
|  | Parkinson’s disease |
|  | Alzheimer’s disease |
| **Problems with the spleen** | Sickle cell anemia |
|  | Removal of spleen |
| **Other chronic health conditions** | Open answer option |
| ^a^These are examples given in the Lifelines questionnaires, participants with chronic diseases beyond these examples were able to mention these as well. | |
