# Supplementary material for: Sex and Gender-Related Differences in COVID-19 Diagnoses and SARS-CoV-2 Testing Practices During the First Wave of the Pandemic: The Dutch Lifelines COVID-19 Cohort Study
Source: J Womens Health (Larchmt). 2021 Dec 16;30(12):1686–92. doi: 10.1089/jwh.2021.0226 (PMC8721498; doi:10.1089/jwh.2021.0226)
Supplement: Supplemental data [file Supp_Appendix4.docx]

**Appendix D: Bivariate analyses**

| **Bivariate analyses, N=74,722.** | | | | | | |  |
| --- | --- | --- | --- | --- | --- | --- | --- |
| **Dependent variable: COVID-19 diagnosis** | | | | **Dependent variable: SARS-CoV-2 PCR test** | | | |
| **Predictor** | | | **OR (95% CI)** | **Predictor** | | | **OR (95% CI)** |
| **Female sex** | | | **1.37 (1.21-1.55)** | **Female sex** | | | **2.04 (1.74-2.41)** |
| **Age** | | | **0.98 (0.98-0.99)** | **Age** | | | **0.97 (0.96-0.97)** |
| **Educational attainment** | | **Low** | 1.00 (ref) | **Educational attainment** | **Low** | | 1.00 (ref) |
|  |  | **Medium** | 1.03 (0.91-1.16) |  | **Medium** | | **0.80 (0.69-0.92)** |
|  |  | **High** | **1.14 (1.01-1.28)** |  | **High** | | **1.47 (1.28-1.69)** |
| **Chronic disease present** | | | **1.18 (1.04-1.35)** | **Chronic disease present** | | | 1.09 (0.88-1.34) |
| **Smoking** | | | 0.86 (0.69-1.07) | **Smoking** | | | 0.88 (0.67-1.17) |
| **Frequent handwashing and use of desinfectant** | | | **1.92 (1.34-2.74)** | **Household members ≤ 18 years** | | | **1.54 (1.29-1.86)** |
| **Social distancing** | | | **2.13 (1.37-3.32)** | **Household members 19-59 years** | | | **1.56 (1.28-1.91)** |
| **Avoidance of public transport** | | | **1.35 (1.17-1.56)** | **Household members ≥ 60 years** | | | **0.45 (0.36-0.57)** |
| **Covering nose and mouth in public** | | | **1.62 (1.36-1.92)** | **Working from home** | | | **0.29 (0.23-0.35)** |
| **Household members ≤ 18 years** | | | 0.98 (0.86-1.11) | **Contact profession** | | **No** | 1.00 (ref) |
| **Household members 19-59 years** | | | 0.88 (0.78-1.00) |  |  | **Yes** | **1.84 (1.53-2.22)** |
| **Household members ≥ 60 years** | | | **0.61 (0.53-0.70)** |  |  | **Yes, in education** | **0.53 (0.36-0.77)** |
| **Working from home** | | | **1.36 (1.20-1.54)** |  |  | **Yes, in healthcare** | **7.61 (6.35-9.11)** |
| **Contact profession** | **No** | | 1.00 (ref) |  |  |  |  |
|  | **Yes** | | **1.58 (1.37-1.83)** |  |  |  |  |
|  | **Yes, in education** | | 1.06 (0.86-1.30) |  |  |  |  |
|  | **Yes, in healthcare** | | **1.39 (1.13-1.72)** |  |  |  |  |
